# An exploration of the potential benefit of extensive listening along with orthography for improving EFL learners’ pronunciation

**DOI:** 10.1186/s40862-022-00132-x

**Published:** 2022-02-18

**Authors:** Nada Almalki, Ghazi Algethami

**Affiliations:** 1 Deanship of Supportive Studies, English Language Center, Taif University, Taif, Saudi Arabia; 2 Department of Foreign Languages, College of Arts, Taif University, Taif, Saudi Arabia

**Keywords:** Listening, Pronunciation, Foreign accent, Teaching, English

## Abstract

Explicit pronunciation instruction has been shown to be effective in improving language learners’ pronunciation, yet it is not clear whether the same can be said about implicit pronunciation instruction. In addition, the role of orthography in improving language learners’ pronunciation is yet to be fully understood. Thus, this research examined the contribution of extensive listening combined with orthography to the improvement of language learners’ pronunciation. Ninety Saudi learners of English were randomly allocated to three groups and offered different types of instructions: (A) extensive listening only; (B) extensive listening with orthography; and (C) a control group. Similar utterances were collected from the three groups before and after the instructions. The utterances were then presented randomly to a group of native English listeners for global foreign accent rating. Small, though non-significant, improvement was observed for both experimental groups. This may be attributed to the relatively short duration of exposing the learners to the aural input. Semi-structed interviews, with a sample of the learners from the experimental groups, showed that they had positive opinions regarding extensive listening with orthography for the improvement of their pronunciation. Further research may use longer period to examine whether extensive listening along with orthography can result in significant improvement of language learners’ pronunciation.

## Introduction

Despite the importance of pronunciation to successful communication, and the findings of recent research indicating the effectiveness of pronunciation instruction, pronunciation is still given the least attention in English language classrooms (e.g., Darcy et al., 2021; Foote et al., 2016). Research into teachers’ cognition in relation to pronunciation teaching has shown that teachers believe in the importance of pronunciation, but lack time, specialized training, confidence, and access to resources for pronunciation instruction (Darcy, 2018; Baker, 2014; Couper, 2017).

Many previous studies have shown that teaching pronunciation explicitly can result in improving learners’ pronunciation (Lee et al., 2015; Thomson & Derwing, 2015). However, in practice, many teachers and learners do not have the required phonetic knowledge to teach and learn pronunciation (Baker, 2014). One way to address this gap in teacher education is to find out whether other ways of teaching pronunciation which do not require such specialized knowledge can be as effective. One such possible way is to extensively expose learners to L2 input in hope that they can improve their pronunciation, as L2 input can play an important role in developing L2 speech (e.g., Flege, 2008, 2018).

Extensive listening is an easily implemented activity in language classrooms. It refers to listening activities in which the learners listen “for an extended period of time while focusing on meaning” for a variety of purposes, one of which is listening for pleasure (Rost, 2013, p.193). There is a growing, but limited, evidence showing that listening can improve learners’ pronunciation (Kissling, 2013; Trofimovich et al., 2009; Tsang, 2019). Due to the large methodological differences between these studies, more studies are still needed to establish whether exposing learners to L2 aural input can result in improving their pronunciation.

In a different respect, there is emerging evidence showing that orthography may also play a facilitative role in L2 pronunciation development (Bassetti, 2008). Combining listening and orthography by using, for example, audio script may enhance learners’ awareness of the discrepancy between the aural and written input (Sicola & Darcy, 2015). In addition, where two L2 phonemic categories (e.g., /p/ and /b/ in English) are assimilated to one phonemic category in the learners’ L1 (/b/ in Arabic), orthography may help the learners discern the phonetic difference. Moreover, presenting L2 learners with a script along with a listening activity can aid comprehension and consequently reduce the learners’ cognitive load, which might in turn help them pay attention to pronunciation (Jones & Plass, 2002). However, research on the effect of orthography on L2 pronunciation is still emerging, and previous studies have produced contradictory results (Bassetti et al., 2015). This is probably due to the differences in the methodologies used and the languages examined in previous studies.

In addition to its potential pedagogical contribution, the current study aims to contribute to the two emerging bodies of literature discussed above by examining the effect of extensive listening along with orthography on the improvement of L2 pronunciation. The current study seeks to address the following questions:

1. Does extensive listening contribute to the improvement of EFL learners’ pronunciation?
2. Does extensive listening along with orthography contribute to the improvement of EFL learners’ pronunciation?

## Literature review

### Pronunciation instruction

The last two decades have witnessed a burgeoning of research examining the effect of explicit instruction on the improvement of L2 pronunciation. Although it is difficult to reach any strong conclusion, the results of previous studies indicate a positive effect of explicit instruction on L2 speech production (Lee et al., 2015; Saito, 2012; Thomson & Derwing, 2015). On the other hand, little is known about whether the same effect would be found when implicit methods of instructions are used. Research on grammar has shown an advantage of explicit instruction over implicit instruction (Spada & Tomita, 2010). However, the case is largely unclear for pronunciation instruction, as most previous studies have only examined explicit instruction (Lacabex & Gallardo-del-Puerto, 2020). The current study focuses on the use of extensive listening as a form of implicit learning for pronunciation development. We follow the definition of implicit learning by Rebuschat (2010):

Implicit learning is the learning process that results primarily in unconscious knowledge, that is, knowledge that is tacit and inaccessible to conscious introspection. Implicit learning generally occurs without the intention to learn and without awareness of what has been learned, that is, learners are often unaware of having acquired knowledge. (p.597).

Recently, a few studies have examined the effect of listening on the improvement of L2 pronunciation (Kissling, 2013; Peltekov, 2020; Trofimovich et al., 2009; Tsang, 2019). The main assumption of these studies is that with enough exposure to quality L2 input, learners would improve their L2 pronunciation. However, it is far from clear how much input is needed for pronunciation development, and which aspect of L2 pronunciation is susceptible to improvement (Saito & Hanzawa, 2018).

Trofimovich et al. (2009) examined the effect of long-term comprehension-based listening on the improvement of learners’ pronunciation. They compared the pronunciation of two young groups of English learners who were of French background. The two groups received different types of instructions; one group was taught in regular English classrooms and the other was only exposed to readings with audio recordings by native speakers. After two years, the two groups were found almost comparable in their pronunciation development. It might be possible to envisage a higher pronunciation accuracy for the reading-listening only group if they had also attended the regular classes in addition to their comprehension-based practice.

Kissling (2013) provided two groups of L2 Spanish learners with different types of computer-delivered teaching modules. One type included phonetic instruction, while the other only included listening and speaking. Both groups showed improvement in their pronunciation of several Spanish sounds. The most noticeable finding in the study was that even the listening only group showed improvement in their pronunciation. The study is only limited to several sounds, and it is not clear whether the learners’ prosody has improved as well. Similarly, Peltekov (2020) examined the effect explicit (phonetic rules) and implicit instruction (listening to native input) on the degree of perceived foreign accent in the speech of German learners. Both types of instructions resulted in small, though nonsignificant, improvement.

Tasng (2019) examined the effect of narrow listening on the English pronunciation of Cantonese L2 learners. Two groups of Cantonese learners of English were asked to listen to audios spoken by native English speakers as homework. Their pronunciation performance was judged against the performance of a comparable control group who received no such homework. The pronunciation of the groups that listened to the audios as homework showed more improvement than the pronunciation of the control group. Because all the learner groups had attended a course on English phonology prior to their participation in the study, it is not clear how the findings of the study can be generalized to the overall ESL population who may lack such specialized knowledge.

### Assessment of second language speech

The assessment of second/foreign language speech is very complex. It entails several interrelated dimensions (accent, comprehensibility, intelligibility, fluency, segmental and prosodic accuracy, etc.). In their seminal work, Munro and Derwing (1995) showed that although foreign accent, comprehensibility (difficulty of processing speech) and intelligibility (actual understating of speech) were correlated, they were partially independent. Previous studies examining the production of L2 speech vary widely in which aspect they focus on. In addition, previous studies have shown that different aspects of speech can be affected differently by pronunciation instruction. For example, suprasegmental instruction was found more successful than segmental instruction in improving learners’ pronunciation (Derwing et al., 1998; Lee et al., 2020). The current study focuses on global foreign accent for reasons discussed in the method section below.

### Orthography and pronunciation

Surprisingly, given its obvious effect on pronunciation, orthography has only recently received systematic, empirical investigation in the field of L2 speech (Bassetti et al., 2015). Previous studies have yielded contradictory results regarding the role of orthography in L2 speech production. While some studies found that orthography has a facilitative role in the production L2 speech (e.g., Erdener & Burnham, 2005; Steele, 2005), others found that it has a detrimental role in producing certain sounds (e.g., Bassetti & Atkinson, 2015; Bassetti, 2017). Most previous studies are limited to single graphemes and sounds. The overall effect of orthography on L2 pronunciation is not yet known.

The current study examines the effect of extensive listening on the pronunciation of Saudi EFL learners, and whether the provision of orthography along extensive listening can carry any added advantage to learners’ pronunciation progress.

## Methods

### Research design

Because of the exploratory nature of the study, a mixed-method approach was used to gather the data for the current study. A quasi-experiment was first conducted and followed by semi-structed interviews. Ninety Saudi EFL learners of English participated in the current study. They were divided into two experimental groups (listening only & listening with orthography) and one control group. The participants’ pronunciation was evaluated by native English listeners before and after the instruction period. A few of the participants from the experimental groups were later interviewed to explore the learners’ perspectives regarding the potential benefit of listening and orthography for pronunciation improvement.

### Settings

Due to the restrictions caused by COVID-19, the study was carried out entirely online via Zoom (a video conferencing software) in the first half of the year 2021 and was closely monitored in every step by the first author. This included both the intervention meetings as well as the pre- and post-tests, which were not possible to be conducted in person.

The intervention meetings lasted six weeks (2 h/per week). During the intervention period, the participants were attending an online English language course (Intermediate level) taken as part of their study plan at a Saudi public university.

### Participants

A total of 90 female Saudi learners of English volunteered to participate in the current study by responding to an online survey using Google Forms. They were of normal hearing, with no history of vision, speech, hearing, or language-processing disorders. A convenience sampling procedure was used to select the participants for the current study. The 90 participants were enrolled in three different classes for the same course, which was an intermediate-level English language course taught by the first author. The same textbook and teaching materials were used in all the three classes. For the current study, the three classes were assigned randomly to three groups: two experimental groups and one control. The two experimental groups received additional listening activities, while the control group did not. While the two experimental groups listened to the same audios, one was also provided with the audios’ scripts. The participants age ranged from 19 to 20. They were studying different majors at Taif University in Saudi Arabia.

### Stimuli preparation and elicitations

Three sentences were designed to elicit the spoken data for the current study (see Appendix 1). Some considerations were taken during the design of the sentences. First, an attempt was made to imbed in the sentences most of the English sounds that have been shown to be difficult for Saudi learners: /p, v, tʃ, ɹ, ŋ, e, æ, ʌ, ɒ, ɔ: & ɜ:/ (Flege & Port, 1980; Altaha, 1995; Ahmad, 2011). Second, we made sure the sentences did not include any unfamiliar word which may not be appropriate for the participants’ proficiency level. In addition, we made sure that the sentences were long enough to include sufficient prosodic information for the raters.

The same utterances were elicited twice from all the participants in the three groups; before and after the instruction/training period. We used Zoom to conduct the pre-and post-tests due to the restrictions caused by COVID-19. The first author met each participant individually. The three sentences were displayed to the participant using Microsoft PowerPoint via the share feature in Zoom. The participant was given two minutes to read the sentences silently, and then listened to them as read by a native British English speaker. The participant was then asked whether she had any questions about any unfamiliar or difficult words in the sentences. In the few cases where the answer was “yes”, the sentence containing the word was played for one more time. None of the participants received any instruction or assistance on how they should pronounce any of the words. Afterwards, the participant was asked to read all the three sentences in a natural way. We used the recording feature in Zoom to record each participant’s rendition of the sentences. Each utterance produced by each participant was then coded and saved into a computer folder for the rating experiment. 540 utterances (90 participants × 3 sentences × 2 times) were collected by the end of the recording sessions.

### Instruction and materials

All the participants in the three groups (the experimental and the control) were attending a general English language course (intermediate level) at Taif University in Saudi Arabia. The course was taught by the first author. The textbook used in the course was one of the English Unlimited Book series by Cambridge University Press. It was designed to teach the four language skills (listening, speaking, reading, and writing) integratively. Apart from the occasional pronunciation tips, the book was not designed to teach pronunciation.

In addition to the regular English classes, the two experimental groups attended 12 evening sessions spread over 6 weeks, where they listened to 9 audiobooks. The audiobooks (see Appendix 2) were chosen from the Oxford Bookworms Series published by Oxford University Press. The topics of the chosen books were familiar to the participants and of interest to them. This was intended to lessen the cognitive load on the part of the participants to allow them to focus on both meaning and pronunciation features (Change & Read, 2007). All the audiobooks were appropriate to the proficiency level of the students. They corresponded to the A2-B1 levels of the Common European Framework of Reference for Languages (Intermediate). In addition, we asked five English language teachers from the community of English language teachers at Taif University to confirm whether the audiobooks were appropriate to the level of the students. They confirmed the appropriateness of the audiobooks to the learners’ levels.

The first author met with the participants in each experimental group twice a week via Zoom. In each session, the participants were reminded of the importance of being in a quiet place. The first author then played the scheduled audiobook and asked the participants to listen carefully. To ensure that the listeners were paying attention to the aural input, we designed an online questionnaire that the students needed to fill in after listening to each audiobook. The questionnaire asked whether the audiobook was difficult, long, or interesting. In addition, a simple comprehension question was added to the questionnaire and a follow-up discussion about the topic was held to engage the students in the listening activities. The participants’ responses to the comprehension questions indicated that they listened to the audiobooks. The listening and orthography group were provided with a printed version of the books and were instructed to track the text while listening to the audiobook. The only difference between the two experimental groups is that one of them were provided with the printed version of the audiobooks.

### Foreign accent rating

Due to the multifaceted and complicated nature of pronunciation assessment, the current study focused only on the ratings of foreign accent as a measure of pronunciation assessment. There are three main reasons why we chose foreign accent over other dimensions of pronunciations (e.g., accuracy of individual sounds and prosodic elements, fluency, intelligibility, and comprehensibility). Firstly, if we had opted to choose more than one dimension, we would have end up with a large number of stimuli that would be difficult to judge, given that we had 540 utterances. Secondly, many English language learners were found to have a desire to reduce their foreign accent, given its negative impact on social and communicative interactions (Derwing, 2003; Timmis, 2002). Thirdly, and more importantly, it is well known that foreign accent correlates with most dimensions of pronunciation assessment (Munro & Derwing, 1995).

All the utterances collected from all the three groups before and after the instruction period were presented to 10 native British English listeners for accentedness rating. The native judges were all above the age of 18 and reported no hearing impairment. They were all paid for their participation.

The utterances were presented randomly, aurally and in written form, to the native listeners online via Qualtrics (https://www.qualtrics.com). The listeners were asked to rate each utterance for the degree of foreign accent on a 9-point Likert scale (1: no foreign accent; 9: strong foreign accent). This scale is reliable and widely used in the literature of second language speech (Southwood & Flege, 1999). In order to avoid listening fatigue on the part of the listeners, the utterances were presented in three sets. Each set consisted of 180 utterances of one sentence. The listeners were given 15 min break after listening to each set. All the listening sessions were closely monitored via Zoom by the first author.

### Semi-structured interviews

To obtain insights from the learners’ perspective on how the listening activities and orthography may have helped them improve their pronunciation, semi-structured interviews were conducted with six participants from each experimental group. To get a representative sample from each group, we selected two from those who received a relatively low degree of foreign accent, two from those who received a moderate degree of foreign accent, and two from those who received a strong degree of foreign accent. All the interviews took place on Zoom and were recorded and transcribed for further analysis. Consent forms were obtained from all the participants.

Two questions were designed to lead the interviews (see Appendix 3). The interviews were conducted in the participants’ native language, Arabic, to ensure their full participation. Each participant was met individually with the first author. All the participants’ responses were recorded, transcribed orthographically, and then translated into English. The researchers then read through the learners’ responses to familiarize themselves with the data and generate ideas about them.

The second phase involved coding the data manually. During this phase, initial codes were produced and organized into meaningful groups. These groups were used to identify the repeated patterns across the data set.

The third phase involved searching for significant main themes in the initial code groups. After identifying the themes, the researchers reviewed them to ascertain their validity and accuracy in relation to the data.

## Results

To measure the inter-rater reliability, a Cronbach’s alpha coefficient was calculated. The coefficient showed a high interclass correlation of 0.94, which indicates a large consistency between the native listeners’ ratings.

Table 1 below shows the means and standard deviations of the foreign accent ratings for all speaker groups:

**Table 1:** Descriptive statistics for pre- and post-tests

Condition	N	Mean	Std. deviation
*Pre-test*			
Control group	30	5.5467	1.44981
Listening group	30	5.8444	1.25177
Listening and orthography	30	5.5589	1.32413
*Post-test*			
Control group	30	5.4000	1.32016
Listening group	30	5.5800	1.19791
Listening and orthography	30	5.2089	1.48060

**Fig. 1:**
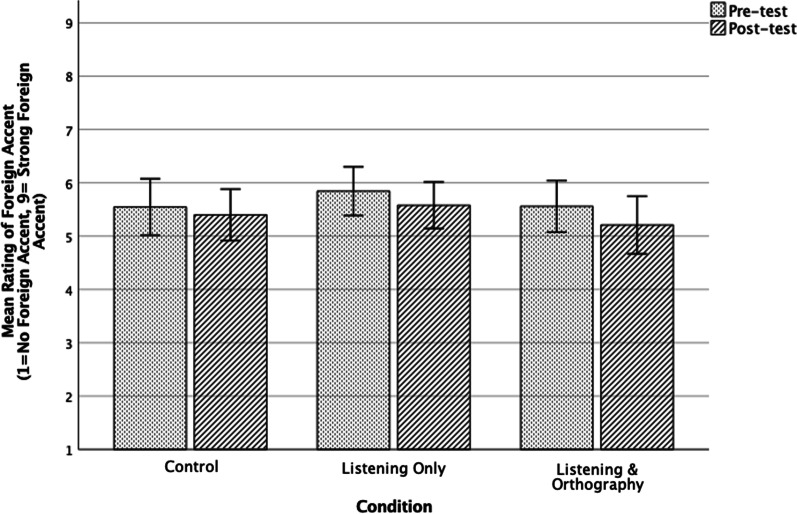
Mean rating scores of foreign accent for all speaking groups in pre- and post-tests

The kurtosis and skewness values for the rating data in both the pre- and post-tests were below 1 (Kurtosis: 0.50 & 0.61; Skewness: 0.64 & 0.67), which indicate that the normality assumption was met. This was also confirmed by the results of the Kolmogorov–Smirnov tests, *p* > 0.05 for both the pre- and post-test data. A two-way repeated measure ANOVA showed a significant main effect of time, *F* (1,87) = 17.41, *p* < 0.05, η^2^ = 0.16. However, there was no significant effect of the interaction between time and group,* F* (1,87) = 0.94, p > 0.05, η^2^ = 0.021. This indicates that there was no significant effect for any of the two types of treatments on the degree of perceived foreign accent. Figure 1 below illustrates the results. The results from the ANOVA are presented below.

In spite of the fact that none of the groups’ accents improved significantly in the post-test, the data revealed slight improvements for the two experimental groups. The control group’s mean scores improved by 0.14 points, whereas the listening only group and the listening with orthography group improved by 0.26 and 0.35 points, respectively. The listening with orthography group yielded the highest improvement.

### Interviews

The qualitative data obtained from the interviews were analyzed thematically. Two main themes emerged from the interviews with the selected participants regarding their opinions about the effectiveness of extensive listening, with or without orthography, on improving their pronunciation. Some key quotes from the learners’ interviews were selected to support the explanation of each theme.

### Awareness-raising, noticing and perception

In general, most of the participants recognized the role of extensive listening in improving their pronunciation. As shared by the participants in the interviews, extensive listening not only helped them pronounce some of the words accurately, but also made them aware of their mistakes and pronunciation problems. For instance, (LO30)^1^ described how the listening activities contributed to her pronunciation improvement:

Listening to the stories helped me a lot in improving my pronunciation. The listening activities made me aware of the correct ways to pronounce some of the words I used to pronounce incorrectly.

Another participant also mentioned that listening to the words and sounds as produced by a native speaker helped her notice how they are actually articulated by native speakers:

The listening activities helped me improve my pronunciation because I can hear the words and sounds produced by a native speaker of English where the articulation is clear and correct. (LO3).

### Sound and spelling

Most of the participants believed that listening to the audios and reading the script at the same time was more helpful to their pronunciation improvement than listening alone. The quotation below exemplifies their preference.

When you listen and read at the same time, you associate the words with their correct pronunciation, and this would help you pronounce them correctly the next time you use them regardless of how they are spelled (LR20).

Although most of the participants recognized the advantage of listening while reading for their pronunciation improvement, one participant from the reading/listening group expressed concern about the simultaneous use of the two modalities:

I suffered as I did not know how to listen and read at the same time. Because of this, I lost track and could not follow. (LR28).

## Discussion

The current study explored the potential use of extensive listening with or without orthography for teaching pronunciation to EFL Saudi learners. Two groups of learners were exposed to the same aural input, except that one of them was also asked to follow a written script of the input. The degree of perceived foreign accent in their speech was assessed before and after the exposure to the input. The two groups showed small, though non-significant, improvement. The learners who received the aural and written input showed slightly higher improvement. Our results are similar to the results obtained by Peltekov (2020), which also found small, but non-significant, improvement in the accent of German learners after they were exposed to native input. On the contrary, Kissling (2013) and Tsang (2019) showed significant improvement gains for L2 pronunciation. However, it should be noted that their studies differed methodologically from our study. Kissling (2013) focused on the pronunciation accuracy of singles phones. In addition, the participants in her study received pronunciation practice with no phonetic rules. All the participants in Tsang (2019) had previous knowledge of English phonology. This might have added some advantage to the listening activities and contributed to their success in improving the learners’ pronunciation. In fact, this is what some of the participants mentioned in the interviews conducted after the experiment.

The results of the students’ interviews in the current study indicated a positive attitude towards the use of listening for pronunciation improvement. This finding is line with the findings of the students’ interviews in Tsang (2019) and Al-Harbi (2019). The participants in these studies recognized the advantage of listening for improving their pronunciation. Given the overall general trend towards pronunciation improvement, and the students’ positive opinions towards the impact of listening on their pronunciation, one can assume that with longer period of extensive exposure to L2 aural input, learners can significantly improve their pronunciation. In fact, Trofimovich et al. (2009) found significant foreign accent reduction in the speech of L2 learners after two years of only comprehension-based language practice.

Previous studies that have examined the effect of orthography on L2 pronunciation improvement have mostly focused on single phones, and have produced contradictory results (Bassetti et al., 2015). This is expected considering the many factors that can affect the production of speech, such as the difference between L1 and L2 phonological and orthographic systems. The current study examined the effect of orthography on overall pronunciation improvement. The results showed slightly higher, but nonsignificant, improvement for the learners who received both the aural and written input. The results from the interviews also supported this finding where the students found the transcript helpful for their pronunciation improvement. However, one student mentioned that listening and following a script at the same time was confusing to her. This could be the result of a difference in learning style (e.g., Oxford et al., 1992).

## Conclusion and further research

The current study sought to address a widely acknowledged and common issue in the language classroom, which is the negligence of pronunciation teaching due to the lack of time, resources, and specialized knowledge on the part of teachers. The potential use of extensive listening, as an easily implemented activity, for pronunciation teaching was explored. The results showed only small, non-significant improvement in the learners’ pronunciation. Taken together with the results from previous studies and the students’ positive opinion towards the use of listening for improving pronunciation, future research may consider examining the effect of extensive listening for longer periods of time on L2 pronunciation improvement. Most previous studies have included some sort of production practice along with the listening activities. The current study did not include any production practice, further research may also examine the effectiveness of extensive listening accompanied with production practice on L2 pronunciation development.

The current study also examined whether orthography, presented as a script along with the listening activity, plays any facilitative role in enhancing the learners’ pronunciation. Although orthography added a small advantage to the pronunciation improvement, we cannot draw any firm conclusion regarding its effect because of the statistically insignificant results. The role of orthography in facilitating the contribution of listening to overall pronunciation improvement is still not clear. Further research may examine the possible facilitative role of orthography given that even the students in the current study recognized its facilitative role.

Methodologically, the current study is limited in two ways. First, it only focuses on one dimension of second language speech, which is foreign accent. Future research may examine the effect of extensive listening with or without orthography on other dimensions of L2 speech, such as comprehensibility, intelligibility, and fluency. Second, we only examined read speech. It may be better and more reflective of students’ actual improvement to examine extemporaneous speech.

At least from the perspectives of students, using extensive listening accompanied with orthography as an extracurricular or homework activity for improving learners’ pronunciation seems worthy of trial by language teachers and learners.

## Data Availability

The data is available upon request by contacting the first author.

## Appendix 1: The English sentences

1. The man standing next to the bus stop is very tall.
2. The girl chose the dress because it was cheaper.
3. She was reading the paper when she saw the text from her sister.

## Appendix 2: The audiobooks

**Table:**

Week	Book levels	Words count	Book tittles
1	Level 2	700	Anne of Green Gables
1	Level 2	700	Alice Adventures in Wonderland
2	Level 2	700	Huckleberry Finn
2	Level 2	700	The Jungle Book
3	Level 2	700	Dracula
3	Level 2	700	The Piano
4	Level 3	1000	The Secret Garden
4	Level 3	1000	The Secret Garden
5	Level 3	1000	Martin Luther King
5	Level 3	1000	Martin Luther King
6	Level 3	1000	A Christmas Carol
6	Level 3	1000	A Christmas Carol

*Level 2 and 3 correspond to the A2-B1 levels in the Common European Framework Refence for Languages

## Appendix 3: Semi-structured Interview Questions

- Do you think extensive listening improved your pronunciation? If yes, how?
- Do you think reading while listening play a role in improving pronunciation? If yes, how?

## Abbreviations

EFL: English as a Foreign language
L2: Second language

## Footnotes

1 LO & LR refer to listening only and listening with orthography, respectively. The number refers to the listeners’ ID.
